# Are the rib fracture score and different computed tomography measures of obesity predictors for mortality in patients with rib fractures? A retrospective cohort study

**DOI:** 10.1007/s00068-020-01483-1

**Published:** 2020-09-06

**Authors:** Thorsten Jentzsch, Valentin Neuhaus, Burkhardt Seifert, Rudolf M. Moos, Hans-Peter Simmen, Christoph E. W. Schmitz, Clément M. L. Werner

**Affiliations:** 1 Department of Traumatology, University Hospital Zurich, University of Zurich, Ramistrasse 100, 8091 Zurich, Switzerland; 2 Department of Orthopaedics, Balgrist University Hospital, University of Zurich, Zurich, Switzerland; 3 Department of Biostatistics, Epidemiology, Biostatistics and Prevention Institute, University of Zurich, Zurich, Switzerland; 4 Medical Directorate, University Hospital Zurich, University of Zurich, Zurich, Switzerland

**Keywords:** Adiposity, Bone, Computed tomography, Diagnosis, Fat mass

## Abstract

**Background:**

There is missing knowledge about the association of obesity and mortality in patients with rib fractures. Since the global measure of obesity (body mass index [BMI]) is often unknown in trauma patients, it would be convenient to use local computed tomography (CT)-based measures (e.g., umbilical outer abdominal fat) as a surrogate. The purpose of this study was to assess (1) whether local measures of obesity and rib fractures are associated with mortality and abdominal injuries and to evaluate (2) the correlation between local and global measures of obesity.

**Materials and methods:**

A retrospective cohort study included all inpatients with rib fractures in 2013. The main exposure variable was the rib fracture score (RFS) (number of rib fractures, uni- or bilateral, age). Other exposure variables were CT-based measures of obesity and BMI. The primary outcome (endpoint) was in-hospital mortality. The secondary outcome consisted of abdominal injuries. Sex and comorbidities were adjusted for with logistic regression.

**Results:**

Two hundred and fifty-nine patients (median age 55.0 [IQR 44.0–72.0] years) were analyzed. Mortality was 8.5%. RFS > 4 was associated with 490% increased mortality (OR_adjusted_ = 5.9, 95% CI 1.9–16.6, *p* = 0.002). CT-based measures and BMI were not associated with mortality, rib fractures or injury of the liver. CT-based measures of obesity showed moderate correlations with BMI (e.g., umbilical outer abdominal fat: *r* = 0.59, *p* < 0.001).

**Conclusions:**

RFS > 4 was an independent risk factors for increased mortality. Local and global measures of obesity were not associated with mortality, rib fractures or liver injuries. If the BMI is not available in trauma patients, CT-based measures of obesity may be considered as a surrogate.

## Introduction

### Background

Rib fractures are commonly (4–12%) found in trauma patients and are associated with a relevant death rate (12%) [1]. Obesity is commonly measured with the body mass index (BMI) and can also be examined by measuring the waist circumference, both of which can predict all-cause mortality [2, 3].

There are studies that have shown that obesity measured with the BMI is a risk factor for peri-traumatic mortality and complications [4], but others have reported that obesity may be protective of certain injuries (e.g., hip fractures) due to a cushioning effect [5]. However, there is a lack of literature about the association of obesity and mortality in patients with rib fractures. One study reported that the BMI is associated with increased incidence rates for multiple rib fractures [6]. An increased incidence of truncal injuries has also been associated with rib fractures [7]. Although multiple rib fractures have been associated with increased mortality in trauma patients and osteoporotic males, there is evidence that rib fractures are not associated with mortality in postmenopausal women and non-stratified patient cohorts [8–13].

The BMI is often not obtainable in trauma patients and the waist circumference is not commonly evaluated. This is due to the facts that severely injured patients often arrive in a supine position, most weight scales would require an upright position, and trauma patients mostly remain immobilized during the initial evaluation. Recently, other computed tomography (CT)-based anthropometric measures of the thorax and abdomen have been introduced as alternative ways to assess obesity [14–18]. Since patients with chest trauma and rib fractures are often evaluated with CT scans, it seems inviting to consider these new tools as alternative ways of obesity assessment and in the prediction of injury risk as well as mortality.

The objectives of this study were to assess whether rib fractures and different measures of obesity are associated with certain truncal injuries and higher mortality. Rib fractures were assessed with the rib fracture score (RFS), which was first described by Easter and is based on the number and side of rib fractures as well as age, [8, 19, 20]. The correlation between the BMI and CT-based measurements of obesity was also assessed. CT-based measurements of adiposity consist of two subcutaneous and two intra-abdominal distances at the level of the subxiphoid and umbilicus.

## Methods

### Study design

From a cohort of 2829 patients that were treated at the authors’ institution, this retrospective cohort study included all inpatients ≥ 18 years with rib fractures that were treated (discharged) at a level 1 trauma center between January 2013 and December 2013. There were no exclusion criteria. The study was approved by the local ethics committee (Kantonale Ethikkommission Zürich, KEK-ZH-Nr.: 2014-0285) without the need for informed consent due to the retrospective nature of this study using a large dataset.

### Exposure and outcome variables

The main exposure variable was the RFS. Other exposure variables were CT-based measures of obesity and placement of a chest tube. The primary outcome variable (endpoint) was in-hospital mortality. Secondary outcomes (endpoints) were abdominal injury with their respective subcategories (liver, spleen, duodenum, colon, kidney, and bladder). A priori confounders consisted of sex and comorbidity.

### Data acquisition and measurements

**Fig. 1:**
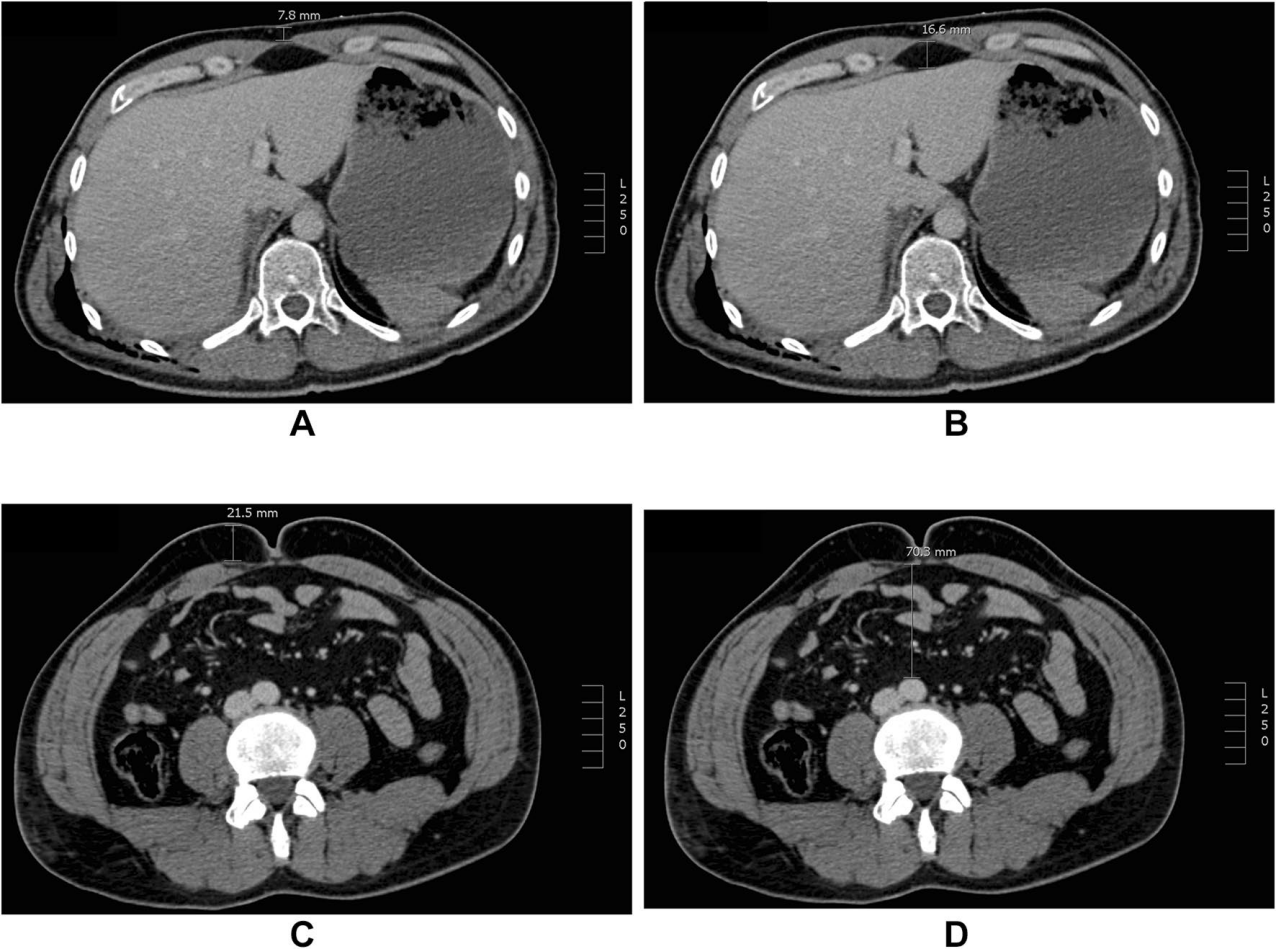
Computed tomography (CT)-based measurements. **a** Subxiphoid outer abdominal fat (SOAF) on an axial plane of a CT scan. **b** Subxiphoid peritoneal fat (SPF) on an axial plane of a CT scan. **c** Umbilical outer abdominal fat (UOAF) on an axial plane of a CT scan. **d** Umbilical visceral fat (UVF) on an axial plane of a CT scan

An independent investigator (senior resident), who was blinded to the data mentioned above, acquired four axial plane CT-based measures of obesity. CT scans were acquired on a dual-source CT scanner (Somatom Definition, Siemens Healthcare, Forchheim, Germany) and images were evaluated using an IMPAX client (version 6.5.5.1544; AGFA HealthCare Corp., Greenville, SC, USA) [16]. The subxiphoid outer abdominal fat (SOAF) was defined as the subcutaneous anterior abdominal wall diameter from the rectus sheath to the cutis just below the xiphoid process [14, 15, 17] (Fig. 1a). The subxiphoid peritoneal fat (SPF) was determined as the intraabdominal distance between the liver and the rectus sheath just below the xiphoid process [14, 15] (Fig. 1b). The umbilical outer abdominal fat (UOAF) was defined as the subcutaneous anterior abdominal wall diameter from the rectus sheath to the cutis at the level of the umbilicus [16] (Fig. 1c). The umbilical visceral fat (UVF) was determined as the distance between the anterior wall of the aorta and the rectus sheath at the level of the umbilicus (Fig. 1d).

The database of the hospital was searched for all patients with at least one rib fracture, coded as S22.31, S22.32, S22.40, S22.41, S22.42, S22.43, S22.44, or S22.5 according to the World Health Organization’s International Statistical Classification of Diseases and Related Health Problems (10th Revision, German Modification, Version 2010) [21, 22]. This search provided several epidemiological patient data, which included sex, age, BMI, the presence of comorbidities (including > 40 diseases, such as coronary heart disease, diabetes mellitus, osteoporosis, neoplastic disease, and depression), death, thoracic injuries, abdominal injuries, and injury severity score (ISS). Thoracic injuries were grouped into isolated rib fracture, and multiple rib fractures of 2, 3 or ≥ 4 ribs. Abdominal injuries consisted of overall intraabdominal injuries, which were further split into injuries of the liver, spleen, duodenum, colon, kidney, and bladder.

The RFS was calculated based on the previously published formula ‘number of rib fractures × side + age category’, where the side was categorized into a binary variable (unilateral = 1 and bilateral = 2) and age was categorized into five categories (< 50 = 0, 50–60 = 1, 61–70 = 2, 71–80 = 3, and > 80 = 4) [8].

### Statistics

Data were tested for distribution with the skewness and kurtosis test for normality. They were non-normally distributed and, therefore, the median and interquartile range (IQR) are provided. To account for some missing data within each category, the absolute number of patients and the corresponding percentage are given in the tables. The Chi-squared test was used for the unadjusted analysis of categorical data. The Wilcoxon rank sum test was employed for continuous data. Spearman rank correlation was used to assess correlations between continuous data. For the main outcome, a logistic regression model was implemented to adjust for the a priori confounders sex and comorbidities and used the Wald test for comparison. For the evaluation of the level of obesity, only sex was included as a confounder, since the number of deaths and the incidence of diseases of interest were low. The BMI and CT-based measurements of obesity were categorized into binary variables based on their respective median. Unadjusted and adjusted odds ratios (OR) and the corresponding 95% confidence intervals (CI) are given. A receiver operating characteristic (ROC) curve was performed to assess potential cut-off points. BMI was also stratified according to the most commonly reported cut-off value in the literature (≥ 30 kg/m^2^) [5]. Due to multiple testing, the significance level was set at 1%. Analyses were performed with Stata (version 13.1/IC; StataCorp, College Station, TX, USA).

## Results

### Participants

Two hundred and fifty-nine patients were analyzed. There were 172 (66.4%) males and the median age was 55.0 (IQR 44.0–72.0) years. The median RFS was 4 (IQR 3–7). One hundred and forty-six (56.4%) patients had comorbidities. The median BMI was 25.1 (IQR 23.1–27.8) kg/m^2^. The median values of the SOAF, SPF, UOAF, and UVF were 9.4 (IQR 23.1–27.8) mm, 12.5 (IQR 8.9–16.2) mm, 19.5 (13.5–25.0) mm, and 59.0 (IQR 44.0–25.0) mm. Thirty-eight (14.7%) patients had an intraabdominal injury, of which 11 (4.3%) were a liver injury. Twenty-two (8.5%) patients died.

### Obesity and mortality

The BMI was higher in males (25.7 [IQR 23.9–29.0] versus [vs] 24.2 [21.1–26.8] kg/m^2^, *p* = 0.002) (Table 1). It was not increased in patients with comorbidity. No association between BMI and mortality (24.4 [IQR 23.5–25.2] vs 26.1 [IQR 22.9–28.0], *p* = 0.59; AUC 0.39 [0.31–0.47]) or abdominal injury were observed. Even when choosing a cut-off point of ≥ 30 kg/m^2^, there were no differences in mortality (0 (0%) deaths for BMI ≥ 30 kg/m^2^ vs 2 (1.6%) deaths for BMI < 30 kg/m^2^, *p* = 1.00), rib fracture score (9 (42.9%) RFS > 4 for BMI vs 50 (40.0%) RFS ≤ 4 for BMI < 30 kg/m^2^, *p* = 0.56) and abdominal injuries (liver: *p* = 0.23, spleen: *p* = 0.21, duodenum: *p* = 1.00, colon: *p* = 1.00, kidney: *p* = 0.38, bladder: *p* = 0.46). Similar results were observed when choosing a cut-off of ≥ 25 kg/m^2^.

**Table 1:** Association of outcome variables and body mass index (BMI)

Variable	Category	Body mass index (BMI) (*n* = 146)				*p* value*
		Median	IQR	*n*	%	
Sex	Female	24.2	21.1–26.8	46	31.5	0.002
	Male	25.7	23.9–29.0	100	68.5	
Age (years)	≤ 55	24.9	23.1–27.8	81	55.5	0.49
	> 55	25.6	23.4–28.1	65	44.5	
Comorbidity	No	24.6	22.5–27.2	65	44.5	0.04
	Yes	26.1	23.9–28.4	81	55.5	
Death	No	25.2	22.9–28.0	144	98.6	0.59
	Yes	24.4	23.5–25.2	2	1.4	
Rib fractures						
Multiple (2)	No	25.0	23.4–28.1	115	78.8	0.78
	Yes	25.5	22.5–27.8	31	21.2	
Multiple (3)	No	25.5	23.5–28.1	121	82.9	0.10
	Yes	24.0	20.8–27.3	25	17.1	
Multiple (≥ 4)	No	25.0	22.5–27.7	89	61.0	0.24
	Yes	25.2	24.0–28.4	57	39.0	
Abdominal injury						
Intraabdominal	No	25.3	23.1–28.1	121	82.9	0.72
	Yes	24.8	23.2–27.8	25	17.1	
Liver	No	25.1	23.1–28.1	138	94.5	0.71
	Yes	25.8	22.4–27.6	8	5.5	
Spleen	No	25.2	22.7–27.8	135	92.5	0.55
	Yes	24.9	23.8–33.2	11	7.5	
Duodenal	No	25.2	23.2–27.8	145	99.3	0.17
	Yes	20.8	20.8–20.8	1	0.7	
Colon	No	25.2	23.1–27.8	145	99.3	0.82
	Yes	24.7	24.7–24.7	1	0.7	
Kidney	No	25.2	23.1–27.8	143	97.9	0.98
	Yes	24.8	20.6–36.8	3	2.1	
Bladder	No	25.3	23.2–28.0	144	98.6	0.24
	Yes	22.7	20.6–24.7	2	1.4	

*Wilcoxon rank sum test

The SOAF was not higher in patients who died (Table 2). It was increased in females, patients > 55 years, and patients with comorbidity. When adjusting for the a priori confounder sex, this lack of association remained (OR_adjusted_ = 2.8, 95% CI 1.03–7.72, *p* = 0.04). Patients with a liver injury did not have different values of SOAF. This association did not change in the logistic regression model (OR_adjusted_ = 0.20, 95% CI 0.04–0.98, *p* = 0.05). An injury of the bladder was associated with lower values of SOAF (3.3 [IQR 2.8–4.1] vs 9.5 [IQR 6.5–14.4], *p* = 0.008). This could not be fitted into a logistic regression model since all cases with a bladder rupture had SPF values ≤ 12.5.

**Table 2:** Association of outcome variables and subxiphoid outer abdominal fat (SOAF)

Variable	Category	Subxiphoid outer abdominal fat (SOAF) (*n* = 248)				*p* value*
		Median	IQR	*n*	%	
Sex	Female	13.1	8.0–18.8	80	32.3	< 0.001
	Male	8.4	5.6–12.3	168	67.7	
Age (years)	≤ 55	7.9	5.5–13.8	128	51.6	0.009
	> 55	10.8	7.4–14.9	120	48.4	
Comorbidity	No	7.8	5.2–11.9	107	43.1	< 0.001
	Yes	10.9	7.4–16.6	141	56.9	
Death	No	9.1	6.2–13.8	226	91.1	0.04
	Yes	13.1	7.8–17.9	22	8.9	
Rib fractures						
Multiple (2)	No	10.0	6.6–14.7	197	79.4	0.07
	Yes	7.8	4.9–13.7	51	20.6	
Multiple (3)	No	10.1	6.5–13.9	205	82.7	0.78
	Yes	8.8	6.2–15.7	43	17.3	
Multiple (≥ 4)	No	8.8	5.0–13.9	139	56.0	0.06
	Yes	10.5	6.9–15.3	109	44.0	
Abdominal injury						
Intraabdominal	No	10.1	6.5–14.6	210	84.7	0.15
	Yes	8.1	5.5–12.1	38	15.3	
Liver	No	9.8	6.5–14.5	237	95.6	0.02
	Yes	6.5	3.9–8.8	11	4.4	
Spleen	No	9.7	6.2–14.5	231	93.1	0.53
	Yes	8.8	6.5–11.8	17	6.9	
Duodenal	No	9.5	6.2–14.4	247	99.6	0.98
	Yes	9.3	9.3–9.3	1	0.4	
Colon	No	9.4	6.4–14.4	247	99.6	0.17
	Yes	4.1	4.1–4.1	1	0.4	
Kidney	No	9.4	6.5–14.3	244	98.4	0.58
	Yes	9.3	4.4–13.6	4	1.6	
Bladder	No	9.5	6.5–14.4	245	98.8	0.008
	Yes	3.3	2.8–4.1	3	1.2	

*Wilcoxon rank sum test

For SPF, no associations were seen with mortality (Table 3). However, lower values were found in males, patients aged > 55 years, and patients with injuries of the bladder. The lack of association between a SPF > 12.5 mm and a liver injury remained in a logistic regression model (OR_adjusted_ = 0.20, 95% CI 0.04–0.94, *p* = 0.042). Again, a logistic regression model for a bladder injury could not be fitted.

**Table 3:** Association of outcome variables and subxiphoid peritoneal fat (SPF)

Variable	Category	Subxiphoid peritoneal fat (SPF) (*n* = 248)				*p* value*
		Median	IQR	*n*	%	
Sex	Female	10.6	8.2–14.5	80	32.3	0.004
	Male	13.2	9.5–17.2	168	67.7	
Age (years)	≤ 55	10.9	7.9–15.1	128	51.6	0.003
	> 55	13.4	10.1–17.2	120	48.4	
Comorbidity	No	12.7	8.2–16.1	107	43.1	0.77
	Yes	12.3	9.3–16.5	141	56.9	
Death	No	12.4	8.8–16.3	226	91.1	0.99
	Yes	13.1	9.9–14.7	22	8.9	
Rib fractures						
Multiple (2)	No	12.8	9.3–16.2	197	79.4	0.13
	Yes	10.6	7.8–15.9	51	20.6	
Multiple (3)	No	12.5	9.2–16.4	205	82.7	0.38
	Yes	12.1	7.9–15.6	43	17.3	
Multiple (≥ 4)	No	12.3	8.1–15.7	139	56.0	0.12
	Yes	12.8	9.5–16.6	109	44.0	
Abdominal injury						
Intraabdominal	No	12.8	9.2–16.4	210	84.7	0.11
	Yes	10.5	7.9–14.4	38	15.3	
Liver	No	12.7	9.1–16.3	237	95.6	0.03
	Yes	9.8	5.9–12.5	11	4.4	
Spleen	No	12.7	9.0–16.3	231	93.1	0.50
	Yes	11.0	8.3–14.3	17	6.9	
Duodenal	No	12.5	8.8–16.2	247	99.6	0.79
	Yes	11.0	11.0–11.0	1	0.4	
Colon	No	12.5	9.0–16.2	247	99.6	0.12
	Yes	5.3	5.3–5.3	1	0.4	
Kidney	No	12.5	8.8–16.2	244	98.4	0.93
	Yes	12.6	9.2–17.5	4	1.6	
Bladder	No	12.6	9.1–16.2	245	98.8	0.009
	Yes	5.3	5.0–6.6	3	1.2	

*Wilcoxon rank sum test

No association was found for UOAF and mortality (Table 4). Lower values of UOAF were associated with a liver injury. This association was weakened when applying the logistic regression model (OR_adjusted_ = 0.21, 95% CI 0.05–1.01, *p* = 0.05).

**Table 4:** Association of outcome variables and umbilical outer abdominal fat (UOAF)

Variable	Category	Umbilical outer abdominal fat (UOAF) (*n* = 242)				*p* value*
		Median	IQR	*n*	%	
Sex	Female	19.9	14.4	79	32.6	0.42
	Male	18.7	13.3–24.6	163	67.4	
Age (years)	≤ 55	20.7	13.5–26.7	126	52.1	0.20
	> 55	18.7	13.7–23.3	116	47.9	
Comorbidity	No	19.5	12.5–24.6	105	43.4	0.50
	Yes	19.5	14.4–25.2	137	56.6	
Death	No	19.5	13.3–25.0	221	91.3	0.64
	Yes	20.2	16.3–23.4	21	8.7	
Rib fractures						
Multiple (2)	No	20.2	14.1–25.1	192	79.3	0.27
	Yes	18.1	11.7–24.7	50	20.7	
Multiple (3)	No	19.5	13.5–25.5	199	82.2	0.98
	Yes	19.9	13.2–24.9	43	17.8	
Multiple (≥ 4)	No	18.8	13.1–24.7	136	56.2	0.21
	Yes	21.0	13.7–26.3	106	43.8	
Abdominal injury						
Intraabdominal	No	20.2	13.9–25.7	205	84.7	0.04
	Yes	16.7	11.7–22.4	37	15.3	
Liver	No	19.9	13.6–25.5	231	95.5	0.010
	Yes	13.9	10.4–18.1	11	4.5	
Spleen	No	19.7	13.6–25.3	226	93.4	0.34
	Yes	16.8	12.0–23.4	16	6.6	
Duodenal	No	19.5	13.5–25.0	241	99.6	0.69
	Yes	16.9	16.9–16.9	1	0.4	
Colon	No	19.5	13.5–25.0	241	99.6	0.42
	Yes	13.9	13.9–13.9	1	0.4	
Kidney	No	19.6	13.6–25.0	238	98.3	0.23
	Yes	11.6	5.3–25.3	4	1.7	
Bladder	No	19.6	13.5–25.2	239	98.8	0.10
	Yes	13.9	3.8–17.6	3	1.2	

*Wilcoxon rank sum test

For UVF, no associations were observed with mortality (Table 5). Higher values were seen in males and patients aged > 55 years. Patients with multiple (≥ 4) rib fractures and liver injuries showed lower values of UVF. In the logistic regression model, this association disappeared (OR_adjusted_ = 1.67, 95% CI 0.99–2.81, *p* = 0.06, OR_adjusted_ = 0.08 95% CI 0.01–0.67, *p* = 0.02, respectively).

**Table 5:** Association of patient characteristics and outcome variables and umbilical visceral fat (UVF)

Variable	Category	Umbilical visceral fat (UVF) (*n* = 242)				*p* value*
		Median	IQR	*n*	%	
Sex	Females	49.5	37.6–63.0	79	32.6	< 0.001
	Males	64.7	49.7–84.0	163	67.4	
Age (years)	≤ 55	57.2	42.1–76.2	126	52.1	0.007
	> 55	62.1	47.6–88.5	116	47.9	
Comorbidities	No	58.7	44.8–80.5	105	43.4	0.42
	Yes	59.3	44.0–82.6	137	56.6	
Death	No	59.3	44.0–81.1	221	91.3	0.89
	Yes	57.9	44.9–78.6	21	8.7	
Rib fractures						
Multiple (2)	No	58.7	44.8–81.2	192	79.3	0.87
	Yes	62.2	42.9–79.8	50	20.7	
Multiple (3)	No	59.7	45.1–81.1	199	82.2	0.08
	Yes	52.9	39.6–76.2	43	17.8	
Multiple (≥ 4)	No	55.8	41.8–77.7	136	56.2	0.010
	Yes	53.6	49.0–82.0	106	43.8	
Abdominal injury						
Intraabdominal	No	59.6	44.9–81.2	205	84.7	0.50
	Yes	56.6	40.4–76.2	37	15.3	
Liver	No	59.7	44.9–81.2	231	95.5	0.006
	Yes	44.0	37.1–56.6	11	4.5	
Spleen	No	59.0	43.9–81.1	226	93.4	0.57
	Yes	61.4	54.6–78.5	16	6.6	
Duodenal	No	59.3	44.7–81.1	241	99.6	0.18
	Yes	34.3	34.3–34.3	1	0.4	
Colon	No	59.3	44.7–81.1	241	99.6	0.13
	Yes	29.6	29.6–29.6	1	0.4	
Kidney	No	59.0	44.0–81.1	238	98.3	0.97
	Yes	65.1	47.2–77.4	4	1.7	
Bladder	No	59.3	44.8–81.1	239	98.8	0.03
	Yes	40.4	29.6–40.8	3	1.2	

*Wilcoxon rank sum test

Rib fractures of two ribs were not associated with any of the measures of obesity (*p* = 0.78 for BMI, *p* = 0.07 for SOAF, *p* = 0.13 for SPF, *p* = 0.27, and *p* = 0.87 for UOAF).

### Levels of obesity

**Fig. 2:**
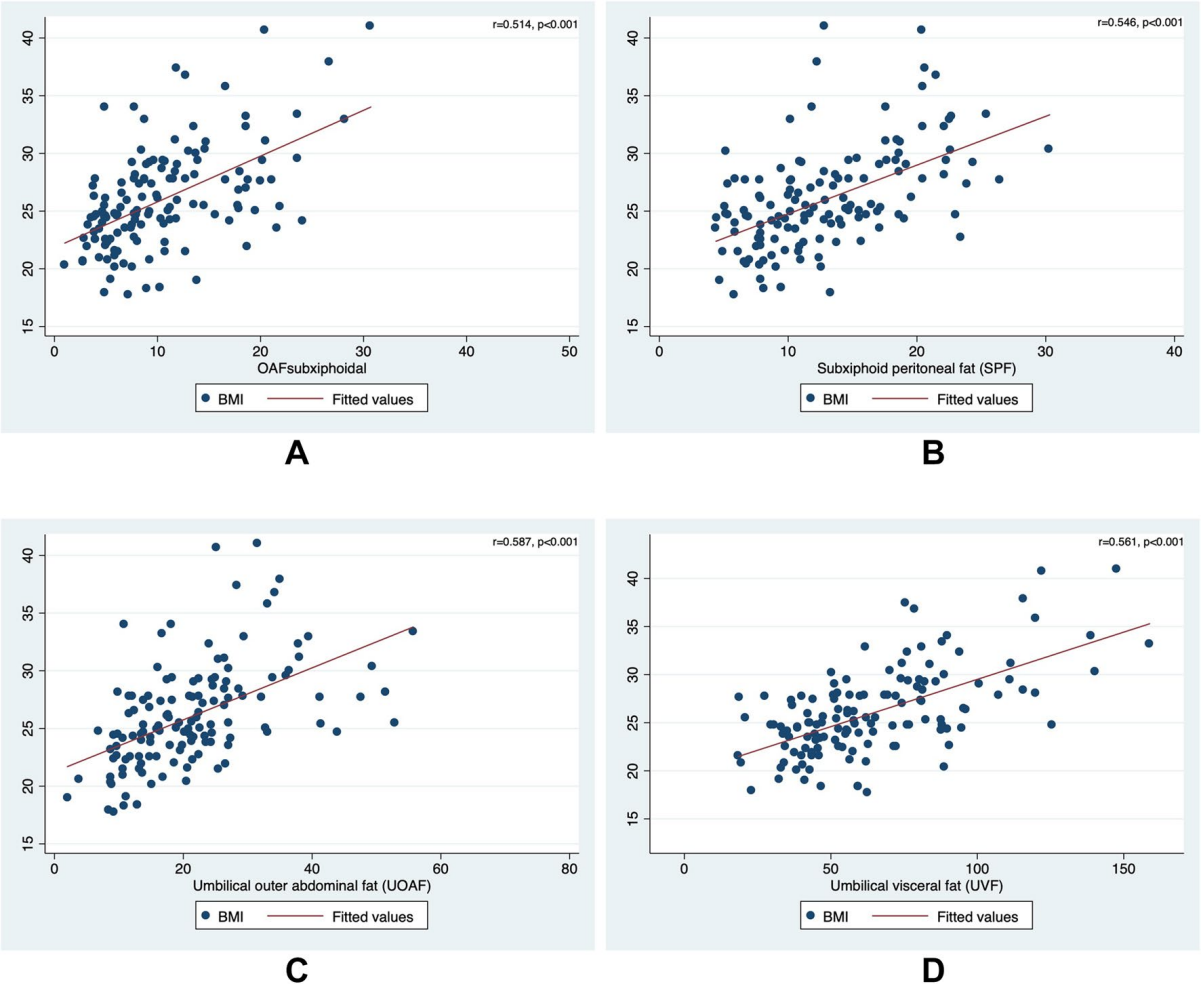
Correlations of the body mass index (BMI) with all computed tomography (CT)-based measurements for the level of obesity. **a** Subxiphoid outer abdominal fat (SOAF). **b** Subxiphoid peritoneal fat (SPF). **c** Umbilical outer abdominal fat (UOAF). **d** Umbilical visceral fat (UVF)

**Fig. 3:**
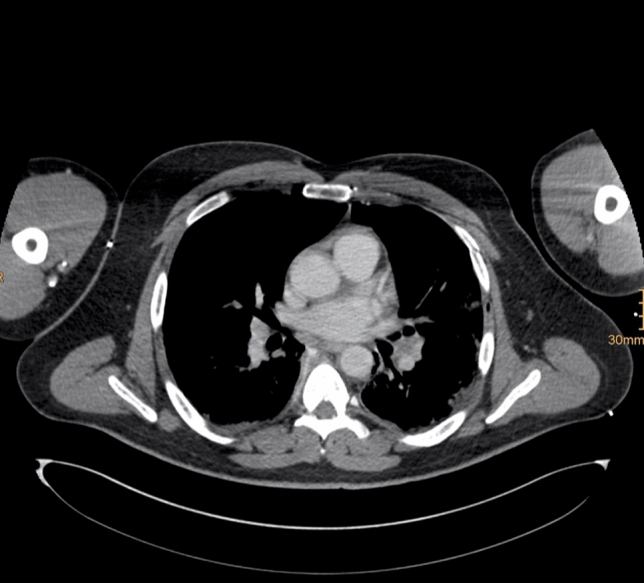
Illustration of a 42-year-old male with a left-sided anterolateral rib fracture and a BMI ≥ 30 kg/m^2^

The BMI showed moderate correlations with all CT-based measurements for the level of obesity (*r* = 0.51 [*p* < 0.001] for SOAF, *r* = 0.55 [*p* < 0.001] for SPF, *r* = 0.59 [*p* < 0.001] for UOAF, and *r* = 0.56 [*p* < 0.001] for UVF) (Table 6; Figs. 2, 3). Both variables for subcutaneous fat revealed a moderate correlation (*r* = 0.64 [*p* < 0.001] for SOAF and UOAF) and both variables for peritoneal fat also manifested a moderate correlation (*r* = 0.53 [*p* < 0.001] for SPF and UVF). Variables for subcutaneous and peritoneal fat showed weak correlations (e.g., *r* = 0.37 [*p* < 0.001] for SOAF and SPF).

**Table 6:** Correlation of different measurement techniques for the level of obesity (*n* = 259)

Variable	Correlation coefficient (*p* value)*				
	BMI	SOAF	SPF	UOAF	UVF
Body mass index (BMI)	1.000	0.514 (< 0.001)	0.546 (< 0.001)	0.587 (< 0.001)	0.561 (< 0.001)
Subxiphoid outer abdominal fat (SOAF)	0.514 (< 0.001)	1.000	0.369 (< 0.001)	0.637 (< 0.001)	0.319 (< 0.001)
Subxiphoid peritoneal fat (SPF)	0.546 (< 0.001)	0.369 (< 0.001)	1.000	0.389 (< 0.001)	0.532 (< 0.001)
Umbilical outer abdominal fat (UOAF)	0.587 (< 0.001)	0.637 (< 0.001)	0.389 (< 0.001)	1.000	0.290 (< 0.001)
Umbilical visceral fat (UVF)	0.561 (< 0.001)	0.319 (< 0.001)	0.532 (< 0.001)	0.290 (< 0.001)	1.000

*Spearman rank correlation coefficient

### Rib fracture score and mortality

**Fig. 4:**
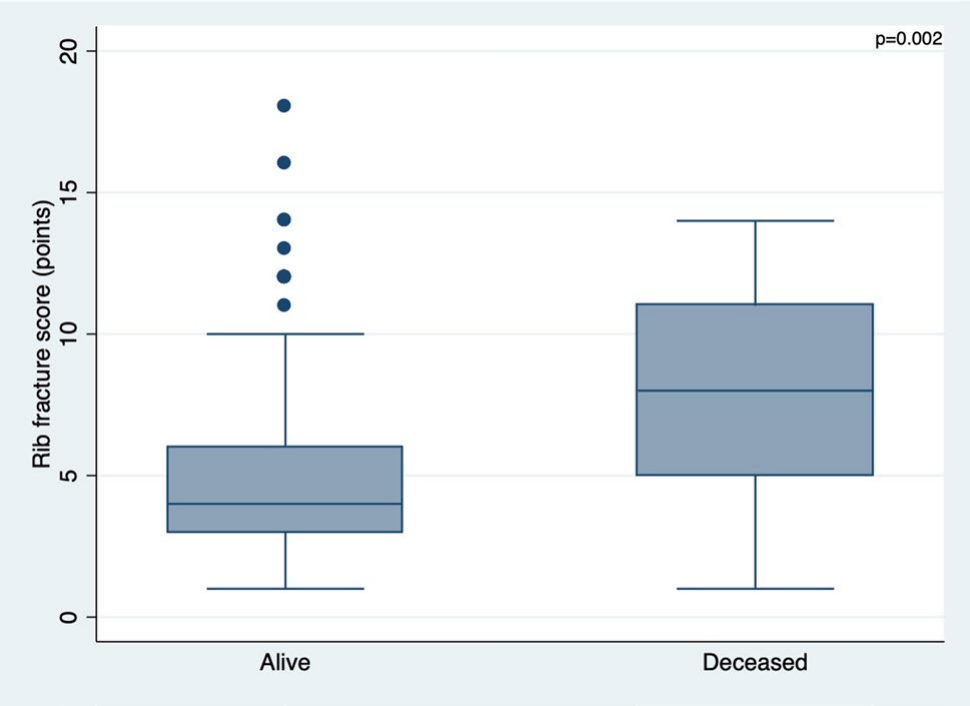
Box plot showing the effect of a RFS > 4 on mortality (*p* value is adjusted for sex and comorbidities)

The unadjusted effect of a RFS > 4 on mortality (OR_crude_ = 5.9, 95% CI 1.9–18.3, *p* = 0.001) remained after fitting a logistic regression model for the a priori confounders sex and comorbidities (OR_adjusted_ = 5.9, 95% CI 1.9–16.6, *p* = 0.002) (Table 7; Fig. 4). The area under the curve was 0.75 (95% CI 0.69–0.80). The chosen cut-off point for mortality was a RFS of 6 (sensitivity of 72.7% and specificity of 67.9%).

**Table 7:** Unadjusted data and logistic regression model for the association of the rib score with mortality (*n* = 259)

Variable	Category	Unadjusted			Adjusted*		
		OR	95% CI	*p* value^†^	OR	95% CI	*p* value^‡^
Rib fracture score (RFS)^§^	≤ 4	1.0 (reference)			1.0 (reference)		
	> 4	5.9	1.9–18.3	0.001	5.9	1.9–16.6	0.002
Sex	Female	1.0 (reference)			1.0 (reference)		
	Male	0.7	0.3–1.7	0.5	0.9	0.3–2.2	0.74
Comorbidities	No	1.0 (reference)			1.0 (reference)		
	Yes	1.4	0.6–3.5	0.47	0.9	0.3–2.3	0.80

*OR* odds ratio, *CI* confidence interval

*Adjusted for all variables given in the table. Sex and comorbidities were considered as a priori confounders

^†^Chi-squared test

^‡^Wald test

^§^Rib fracture score (RFS): number of rib fractures × side + age category (where side: unilateral = 1 and bilateral = 2; age category: < 50 = 0, 50–60 = 1, 61–70 = 2, 71–80 = 3, and > 80 = 4) [17]

## Discussion

One of the main findings of this study about patients with rib fractures is the association between a RFS > 4 and mortality. The global and local measures of obesity were not associated with mortality, rib fractures of liver injuries. The BMI correlated moderately well with the CT-based measures of obesity.

The logistic regression model successfully controlled for sex and comorbidities. It would have been desirable to control for ISS, which had a median value of 25 (IQR 17.0–41.0). However, ISS was only available for 101 patients. This would have led to a substantial reduction of the sample size (from *n* = 259 to *n* = 101) and loss of power. Nonetheless, addition of the ISS to the logistic regression model would have reduced the effect of the RFS > 4 on mortality in the reduced sample (OR_adjusted_ = 4.0, 95% CI 0.9–16.9, *p* = 0.07). Therefore, future studies may include the ISS as a potential confounder in their logistic regression analysis.

Previous studies found that rib fracture patients had higher risks of adverse outcomes, such as pneumonia and that increasing numbers of rib fractures were associated with mortality [7–9]. In 64,750 rib fracture patients from the National Trauma Data Bank (NTDB), the overall death rate was 10% [9]. When stratified according to the number of rib fractures, patients with only one rib fracture had a mortality of 5.8%, while those with five rib fractures had a mortality of 9.9%, and those with ≥ 8 rib fractures had a mortality of 34.4%. This is in line with the presented results since an increased RFS > 4 was associated with higher mortality. Contrarily, 2 recent retrospective studies, which included 594 and 1272 patients with rib fractures, did not observe an influence on morbidity in the intensive care unit (ICU) and mortality [12, 13]. This may be explained by the adjustment for confounders in the present study. Furthermore, other scores than the RFS have been proposed, but the RFS score seems to be a useful and easy tool to account for the severity of rib fractures taking into account the patient’s age [19].

A population-based cohort study of men ≥ 65 years showed that multiple rib fractures were more common in obese patients (relative risk = 4.0, 95% CI 1.2–13.5) [6]. In contrast, this association was not observed in postmenopausal women [11]. In the present study, no measures of obesity were associated with rib fractures. Therefore, the level of obesity and local fat does neither seem protective, nor a risk factor for rib fractures.

The BMI and waist circumferences are valuable tools for obesity, but they do lack a precise discernment of fat from other tissue as well as limited accuracy and reproducibility, respectively [17]. A large collaborate report of 57 prospective studies stated that it can be used as a predictor of all-cause mortality [2]. The survival was reduced by up to 4 years if > 30–35 kg/m^2^ and up to 10 years if > 40–45 kg/m^2^. A recent systematic review and meta-analysis by Kinder et al. reported the effect of obesity on Orthopaedic Trauma patients [4]. In 379,333 patients, complications were more common in obese patients [OR for complications of 2.32 (23% complications if BMI ≥ 30 versus 14% if BMI < 25)]. Obesity was also associated with higher mortality, more infections, non-union tibial fractures, and thrombosis. On the other hand, another meta-analysis suggested the “obesity paradox” indicating that obesity may provide some protection against certain injuries due to a cushioning effect and higher regional bone mineral density, such as hip fractures [5]. In 3,126,313 patients, obese patients showed significantly decreased hip fracture risk. Other studies have concluded that obesity does not have a protective effect against fractures [23, 24]. Our study adds to these findings in that there was no observed difference in mortality, rib fracture severity, and abdominal injuries between obese and non-obese patients. Theoretically, BMI may not be an ideal parameter for the “obesity paradox”. In another prospective investigation of a large cohort, the waist circumference was also associated with increased overall mortality [3]. In their study, the correlation between the BMI and waist circumference was very strong (*r* = 0.85). The reason for only moderate correlations with the BMI is difficult to explain. Potentially, the supine position and location of measurements at the subxiphoid and umbilical level may have played a role. One option to overcome this limitation in the future may be to use volumetric measurements instead of distances [18]. In a trauma setting, obtaining the BMI at the initial stage does not seem very feasible because patients are usually in a supine position and measuring the weight and height would be complicated. The waist circumference could be measured more easily, but according to our knowledge, this is not routinely done. This could be attributed to the fact that the focus is set on essential investigations in the resuscitation room according to the algorithm of the advanced trauma life support (ATLS). Evaluating CT-based measures for obesity is a handy option for easy assessment at any time. These could also be studied using ultrasonography [14]. In a study of 26 non-obese patients, strong correlations as well as excellent intra- and interobserver agreement were found between CT- and ultrasonography-based measurements for subcutaneous and visceral measurements [17].

## Conclusions

In this patient cohort, a RFS > 4 was an independent risk factors for increased mortality. Global and local measures of obesity were not associated with mortality, rib fractures or liver injuries. If the BMI is not available in trauma patients, CT-based measures of obesity may be considered as a surrogate for further evaluation.
